# Activity of 25-Hydroxylase in Human Gingival Fibroblasts and Periodontal Ligament Cells

**DOI:** 10.1371/journal.pone.0052053

**Published:** 2012-12-12

**Authors:** Kaining Liu, Huanxin Meng, Jianxia Hou

**Affiliations:** Department of Periodontology, Peking University School and Hospital of Stomatology, Beijing, China; University of Tennessee, United States of America

## Abstract

**Background:**

We previously demonstrated that 25-hydroxyvitamin D_3_ concentrations in gingival crevicular fluid are 300 times higher than those in the plasma of patients with aggressive periodontitis. Here we explored whether 25-hydroxyvitamin D_3_ can be synthesized by periodontal soft tissue cells. We also investigated which of the two main kinds of hydroxylases, CYP27A1 and CYP2R1, is the key 25-hydroxylase in periodontal soft tissue cells.

**Methodology/Principal Findings:**

Primary cultures of human gingival fibroblasts and periodontal ligament cells from 5 individual donors were established. CYP27A1 mRNA, CYP2R1 mRNA and CYP27A1 protein were detected in human gingival fibroblasts and periodontal ligament cells, whereas CYP2R1 protein was not. After incubation with the 25-hydroxylase substrate vitamin D_3_, human gingival fibroblasts and periodontal ligament cells generated detectable 25-hydroxyvitamin D_3_ that resulted in the production of 1α,25-dihydroxyvitamin D_3_. Specific knockdown of CYP27A1 in human gingival fibroblasts and periodontal ligament cells using siRNA resulted in a significant reduction in both 25-hydroxyvitamin D_3_ and 1α,25-dihydroxyvitamin D_3_ production. Knockdown of CYP2R1 did not significantly influence 25-hydroxyvitamin D_3_ synthesis. Sodium butyrate did not influence significantly CYP27A1 mRNA expression; however, interleukin-1β and *Porphyromonas gingivalis* lipopolysaccharide strongly induced CYP27A1 mRNA expression in human gingival fibroblasts and periodontal ligament cells.

**Conclusions:**

The activity of 25-hydroxylase was verified in human gingival fibroblasts and periodontal ligament cells, and CYP27A1 was identified as the key 25-hydroxylase in these cells.

## Introduction

Vitamin D plays an important role in the regulation of bone metabolism and immunological reactions [1], [2]. In humans, vitamin D, in the form of vitamin D_3_, is derived from dietary sources or made from 7-dehydrocholesterol in the skin by exposure to ultraviolet rays [3], [4], [5], [6], [7]. Then, vitamin D_3_ is metabolized by two-step hydroxylations: first 25-hydroxylation in the liver to form 25-hydroxyvitamin D_3_ (25OHD_3_), the major circulating metabolite of vitamin D_3_, followed by 1,α-hydroxylation in the kidney to form 1α,25-dihydroxyvitamin D_3_ (1,25OH_2_D_3_), the biologically active metabolite of vitamin D_3_ [6], [7].

In the early years of biochemical research, a mitochondrial cytochrome P450 (CYP27A1), an important enzyme in the bile acid synthesis pathway [8], [9], was demonstrated to be 25-hydroxylase. Afterwards, Cheng et al. identified a microsomal cytochrome P450 (CYP2R1) with vitamin D 25-hydroxylase activity [10], [11]. In addition, other cytochrome P450 enzymes, such as CYP2C11, CYP2D25, CYP3A4 and CYP2J2, were all identified as vitamin D 25-hydroxylases [12], [13], [14], and the two most active 25-hydroxylases were found to be CYP27A1 and CYP2R1 [10]. It was reported that CYP27A1 was the more abundant 25-hydroxylase in the liver [10], [15]. However, mutations in human and mouse genes encoding CYP27A1 protein influenced bile acid synthesis, but had no consequence on vitamin D metabolism [15], [16], [17], [18]. Thus, the question as to which of these proteins is the key 25-hydroxylase in the liver remains controversial. In addition, it was reported that, besides the liver, there are extra-hepatic sites of 25OHD_3_ synthesis, including the skin [7], [19], [20], [21], prostate [22], [23], macrophages [24], [25], [26], and endothelial cells [24].

Human gingival fibroblasts (hGF) and human periodontal ligament cells (hPDLC) are two kinds of periodontal fibroblasts and are important components of periodontal soft tissues. Our previous study demonstrated that local 25OHD_3_ levels in gingival crevicular fluid were about 300 times higher than that in the plasma of patients with aggressive periodontitis [27], [28]. Since there is abundant 25OHD_3_ around periodontal soft tissues, it was hypothesized that hGF and hPDLC have 25-hydroxylase activity, and can synthesize 25OHD_3_. The objective of this study was to test this hypothesis.

## Results

**Figure 1:**
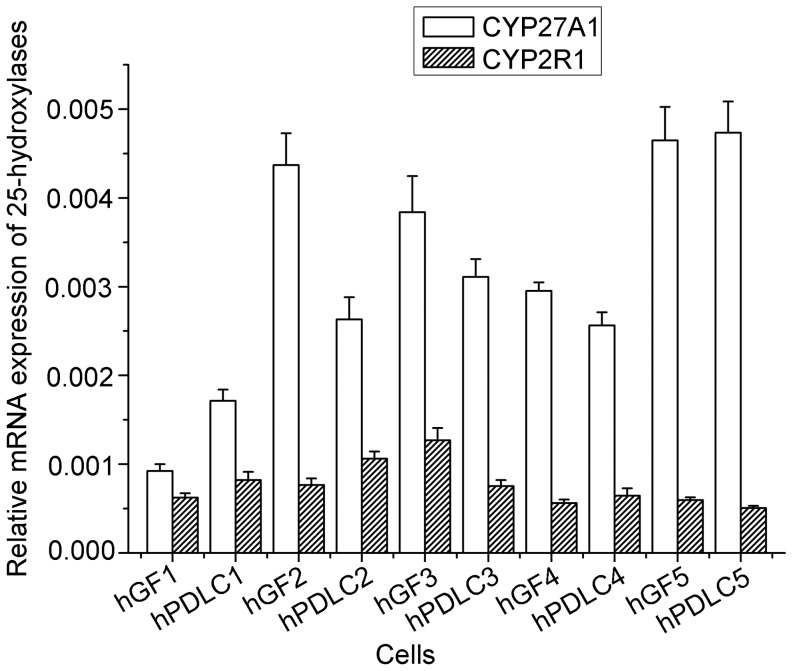
Expression of CYP27A1 and CYP2R1 mRNA in hGF and hPDLC. Expression of CYP27B1 mRNA was detected by real-time PCR in hGF and hPDLC from all five donors (donors are numbered 1–5). The expression levels of CYP27A1 and CYP2R1 mRNA were not significantly different in the two kinds of cells. The data are presented as the mean ± SD.

**Figure 2:**
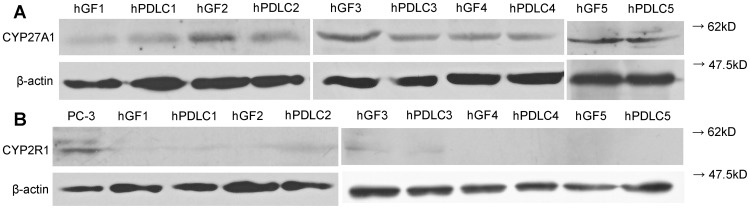
Protein expression of CYP27A1 and CYP2R1 in hGF and hPDLC. Protein expression of CYP27A1 was detected by Western blot in hGF and hPDLC from all five donors (donors are numbered 1–5). Protein expression of CYP2R1 was detected by Western blot in PC-3 cells, which were used as a positive control, but was not detected in hGF and hPDLC. β-actin was used as an internal control.

CYP27A1 and CYP2R1 mRNA were detected in all the cells of the five donors, and no significant difference was found between the mRNA levels in hGF and hPDLC (Fig. 1). CYP27A1 protein was also detected in all cells of the five donors, whereas CYP2R1 was not detected, with the premise that anti-CYP2R1 antibody was able to recognize the protein in PC-3 cells, which were used as a positive control (Fig. 2). This indicated that CYP27A1 might be the key 25-hydroxylase in hGF and hPDLC.

**Figure 3:**
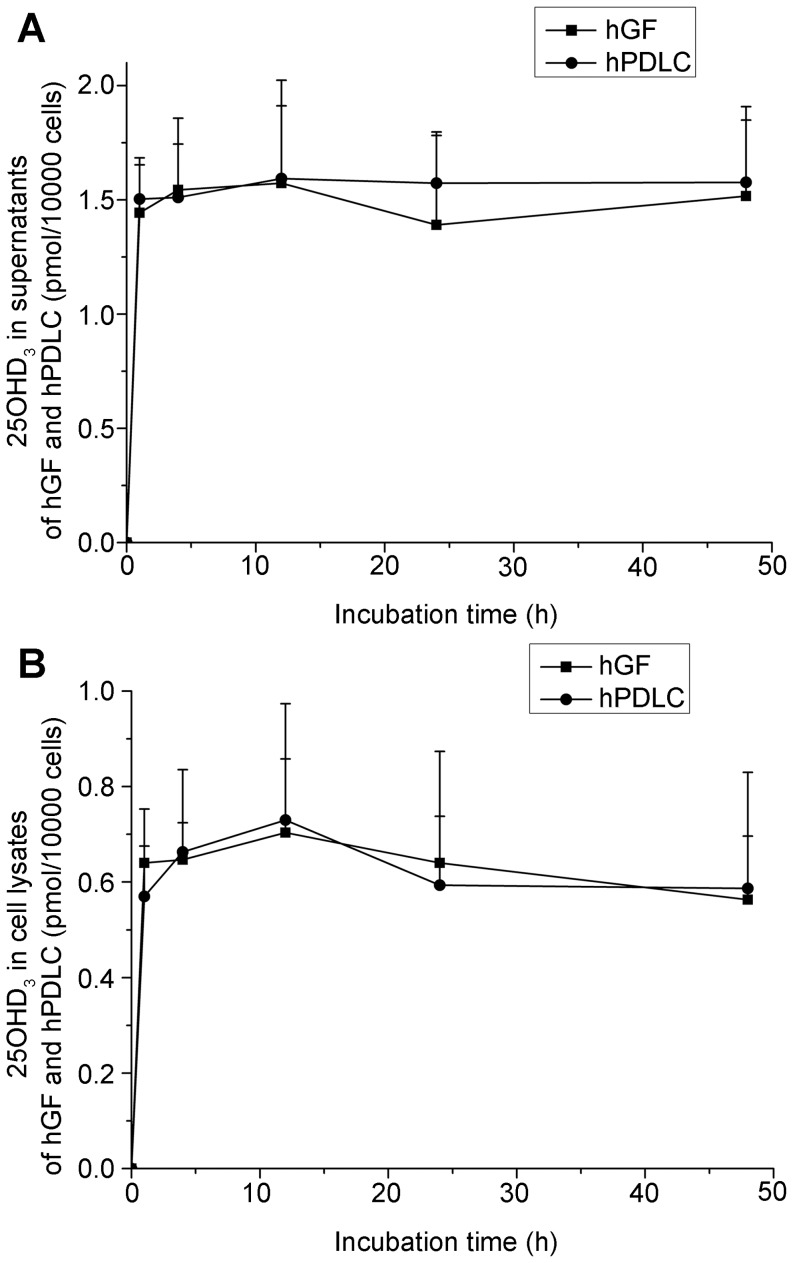
Activity of 25-hydroxylases in hGF and hPDLC. hGF and hPDLC from donors 2, 4 and 5 were incubated with 1000 nM vitamin D_3_ for the times indicated, and the production of 25OHD_3_ was determined in supernatants(A) and cell lysates (B). After incubation, the production of 25OHD_3_ was detected. The amount of 25OHD_3_ generated was not significantly different between hGF and hPDLC. The data are presented as the mean ± SE.

After confirming the expression of 25-hydroxylase in hGF and hPDLC, the function of 25-hydroxylase was investigated. Whereas 1000 nM vitamin D_3_ did not have a significant cytotoxic effect on any of the cells within 48 h, hGF and hPDLC generated 25OHD_3_ in response to vitamin D_3_ (Figs. 3A, B). The fact that extra- and intracellular 25OHD_3_ was generated in the presence of vitamin D_3_ provides direct and convincing evidence of the existence of 25-hydroxylase in hGF and hPDLC. At all time points, there was no significant difference in the levels of intracellular and extracellular 25OHD_3_ between the two cell types.

**Figure 4:**
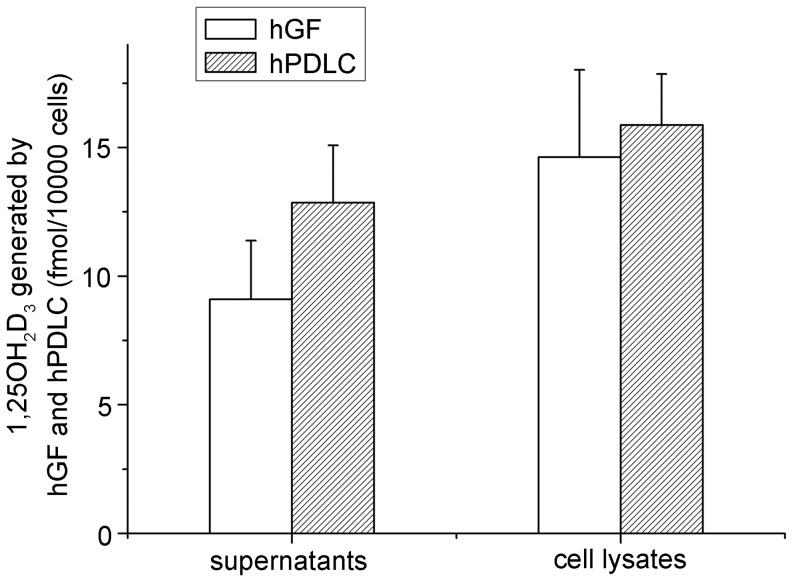
1,25OH_2_D_3_ generation by hGF and hPDLC. hGF and hPDLC from donors 2, 4 and 5 were incubated with 1000 nM vitamin D_3_ for 48 h, and the production of 1,25OH_2_D_3_ was determined in supernatants and cell lysates. The amount of 1,25OH_2_D_3_ generated was not significantly different between hGF and hPDLC. The data are presented as the mean ± SE.

Additionally, exposure to vitamin D_3_ also resulted in the synthesis of 1,25OH_2_D_3_ in hGF and hPDLC (Fig. 4). The observation that hGF and hPDLC could synthesize 1,25OH_2_D_3_ when exposed to 25OHD_3_ [29] is further evidence of 25-hydroxylase activity in hGF and hPDLC.

**Figure 5:**
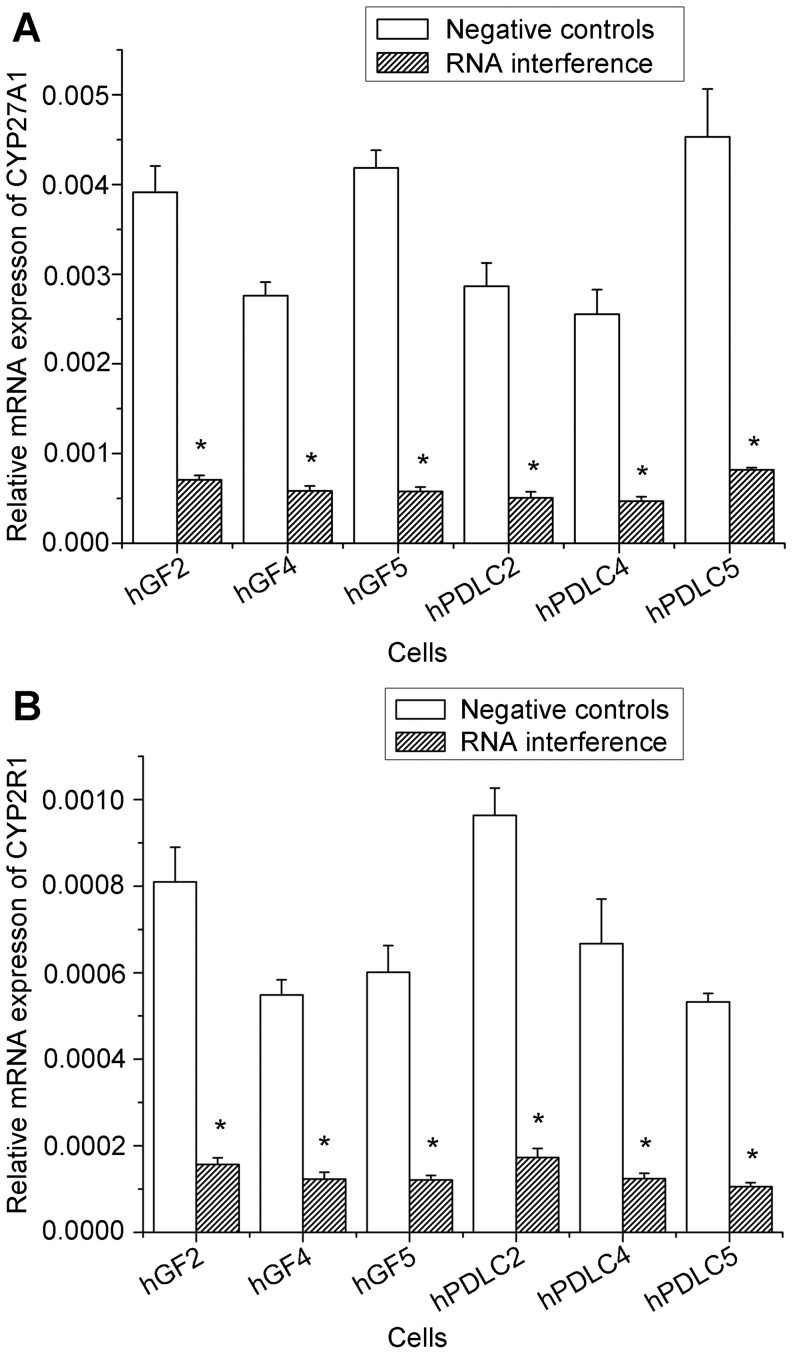
The efficiency of RNA interference against CYP27A1 and CYP2R1. hGF and hPDLC from donors 2, 4 and 5 were transfected with a siRNA oligonucleotide for CYP27B1, a siRNA oligonucleotide for CYP2R1, or a non-silencing control. Using real-time PCR as a measure, the efficiency of RNA interference against CYP27A1 and CYP2R1 was over 70% in hGF and hPDLC. The data are presented as the mean ± SD. * denotes difference from negative controls (*p*<0.05).

**Figure 6:**
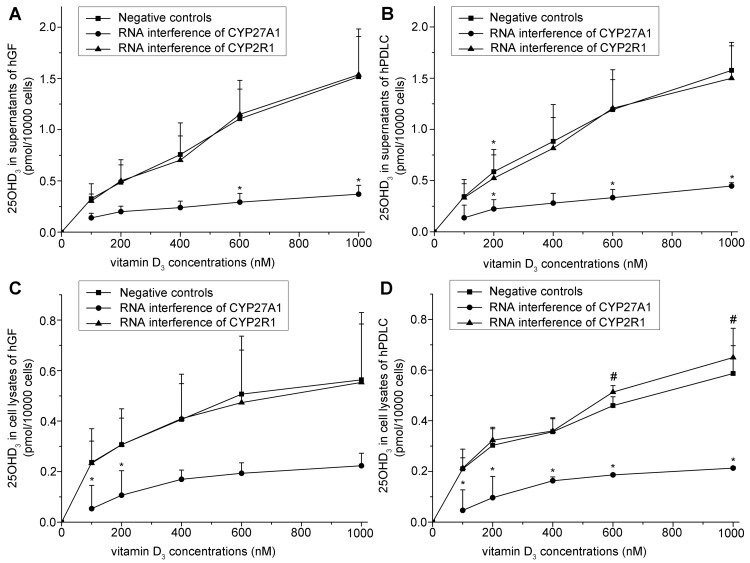
Effect of knockdown of 25-hydroxylases on 25OHD_3_ generation. hGF and hPDLC from donors 2, 4 and 5 were treated with vitamin D_3_ at various concentrations indicated in the figure for 12 h after transfection with a siRNA oligonucleotide for CYP27A1, a siRNA oligonucleotide for CYP2R1, or a non-silencing control. 25OHD_3_ production was measured in supernatants of hGF (A), supernatants of hPDLC (B), cell lysates of hGF (C), and cell lysates of hPDLC (D). When CYP27A1 or CYP2R1 was not knocked down, the production of 25OHD_3_ increased with an increasing concentration of 25OHD_3_. When CYP27A1 was knocked down in hGF and hPDLC, the generation of 25OHD_3_ decreased significantly compared to when CYP27A1 was not knocked down. When CYP2R1 was knocked down in hGF (A, C), the generation of 25OHD_3_ was not significantly different from that when CYP2R1 was not knocked down. When CYP2R1 was knocked down in hPDLC (B, D), the generation of 25OHD_3_ was only slightly different at some time points from that when CYP2R1 was not knocked down. The data are presented as the mean ± SE. * hGF or hPDLC generated significantly less 25OHD_3_ with the same amount of added vitamin D_3_ when CYP27A1 or CYP2R1 was knocked down (*p*<0.05). # hGF or hPDLC generated significantly more 25OHD_3_ with the same amount of added vitamin D_3_ when CYP27A1 or CYP2R1 was knocked down (*p*<0.05).

**Figure 7:**
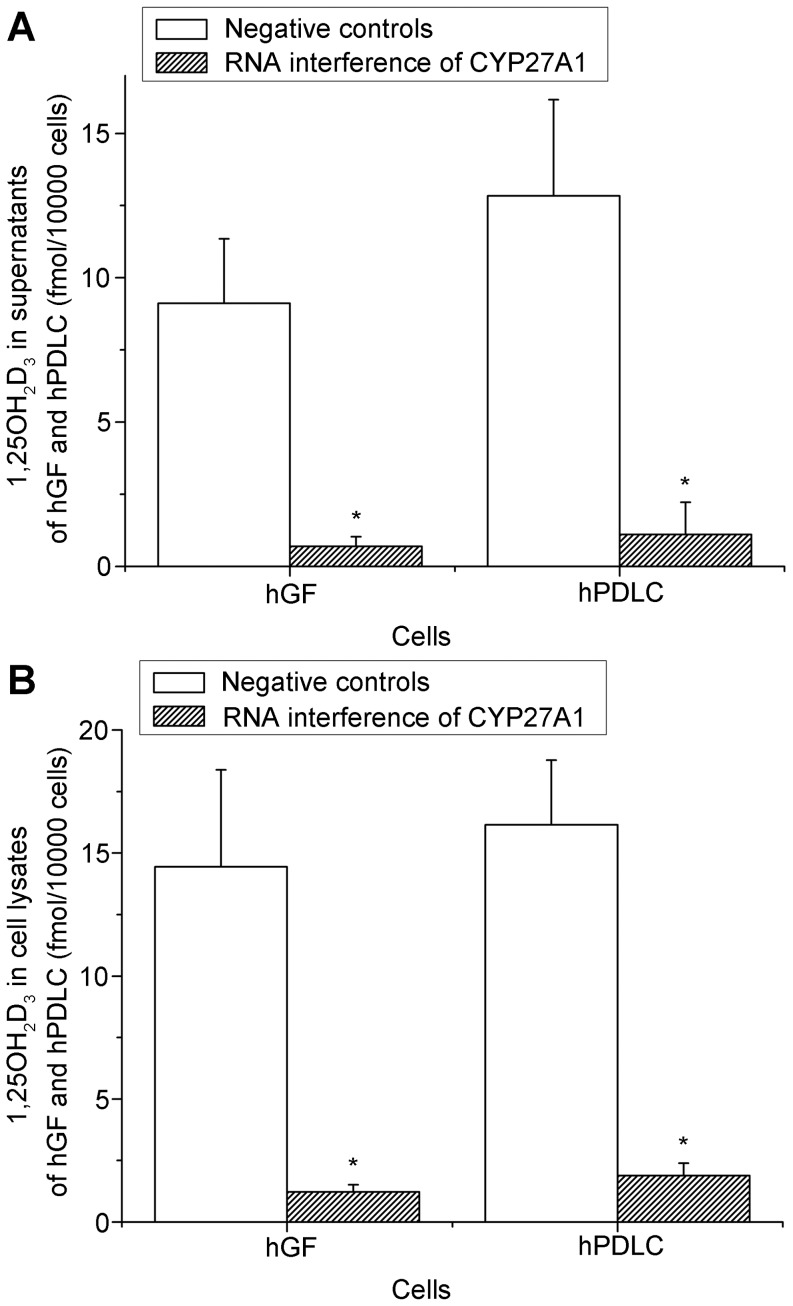
The effect of 25-hydroxylase knockdown on 1,25OH_2_D_3_ generation. hGF and hPDLC from donors 2, 4 and 5 were treated with 1000 nM vitamin D_3_ for 48 h after transfection with a siRNA oligonucleotide for CYP27A1 or a non-silencing control, and 1,25OH_2_D_3_ production was measured in supernatants(A) and cell lysates (B). When CYP27A1 was knocked down, the generation of 1,25OH_2_D_3_ decreased significantly compared to when CYP27A1 was not knocked down. The data are presented as the mean ± SE. * hGF or hPDLC generated significantly less 1,25OH_2_D_3_ with 1000 nM vitamin D_3_ when CYP27A1 was knocked down (*p*<0.05).

Based on the above direct evidence for 25-hydroxylase activity in hGF and hPDLC, we examined the effect of 25-hydroxylase knockdown. The efficiency of RNA interference against both CYP27A1 and CYP2R1 was both over 70% (Fig. 5). The generation of 25OHD_3_ increased with increasing vitamin D_3_ concentrations, but dropped significantly when CYP27A1 was knocked down using specific siRNA (Figs. 6A–D). However, knockdown of CYP2R1 did not significantly influence 25OHD_3_ generation by hGF (Figs. 6A, C), and only slightly influenced 25OHD_3_ generation by hPDLC (Figs. 6B, D). These results suggest that CYP27A1 might be the key 25-hydroxylase in hGF and hPDLC. In addition, knockdown of CYP27A1 resulted in a significant reduction of 1,25OH_2_D_3_ generation (Figs. 7A–B). This is additional evidence for the activity of CYP27A1 as the 25-hydroxylase in hGF and hPDLC.

**Figure 8:**
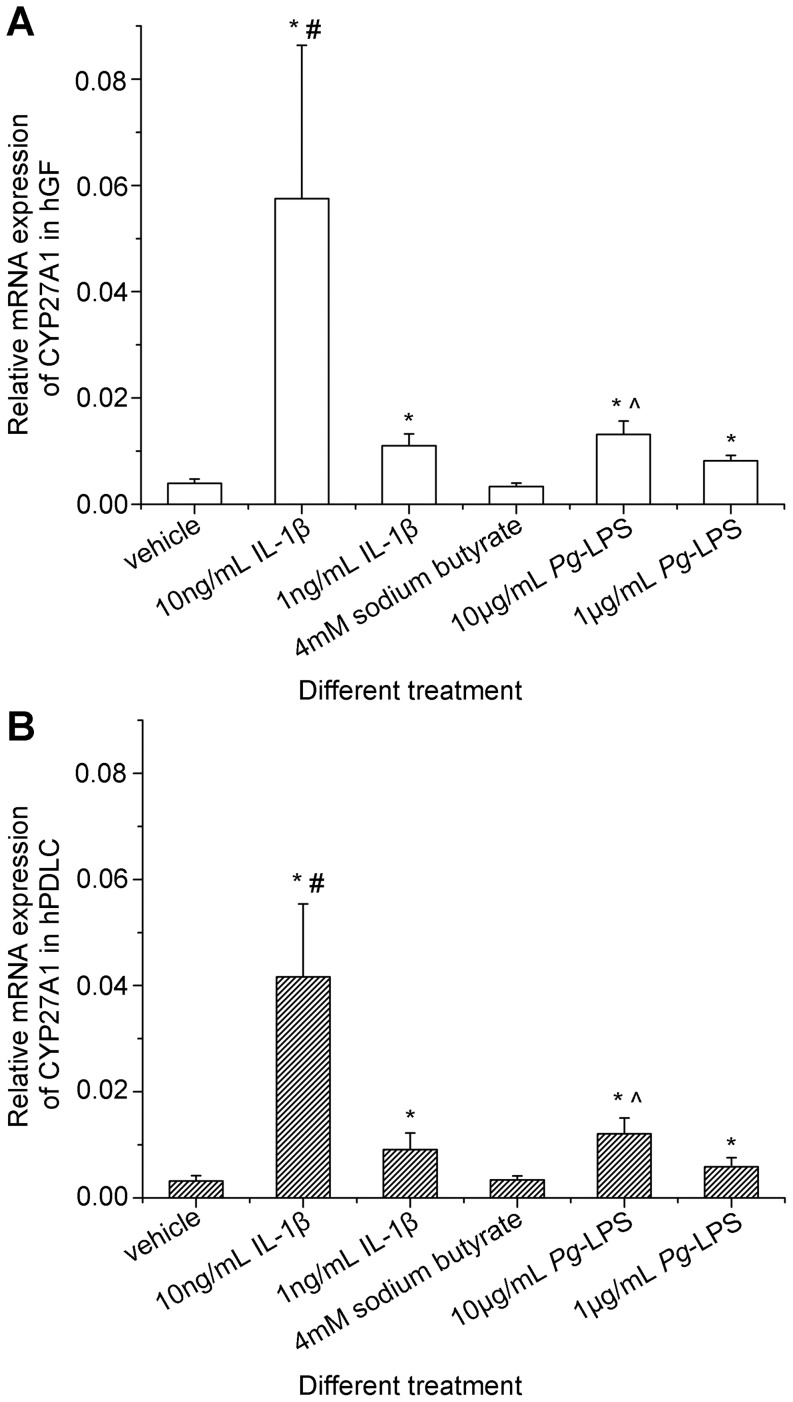
Preliminary investigation of CYP27A1 regulation by inflammatory stimuli in hGF and hPDLC. hGF and hPDLC from donors 2, 3, 4 and 5 were stimulated with different treatments indicated in the figure for 24 h, and CYP27A1 mRNA expression was determined by real-time PCR. IL-1β and *Pg*-LPS significantly up-regulated CYP27A1 mRNA expression and the higher dose of IL-1β or *Pg*-LPS raised higher CYP27A1 mRNA up-regulation in both hGF and hPDLC. Sodium butyrate did not significantly influence CYP27A1 mRNA expression. Additionally, the characteristics of CYP27A1 regulation in hGF and hPDLC were not significantly different. The data are presented as the mean ± SE. * CYP27A1 mRNA expression was significantly different from that of the vehicle group (*p*<0.05). # CYP27A1 mRNA expression was significantly different from that of the 1 ng/mL IL-1β group (*p*<0.05). ^∧^ CYP27A1 mRNA expression was significantly different from that of the 1 µg/mL *Pg*-LPS group (*p*<0.05). IL-1ß : interleukin-1β. *Pg*-LPS: *Porphyromonas gingivalis* lipopolysaccharide.

After the comprehensive confirmation of 25-hydroxylase activity in hGF and hPDLC, and the verification of CYP27A1 as the key 25-hydroxylase, the regulation of CYP27A1 in hGF and hPDLC was investigated. Interleukin-1β (IL-1β) and *Porphyromonas gingivalis* lipopolysaccharide (*Pg*-LPS) strongly induced CYP27A1 expression (Fig. 8). Additionally, dose-dependent increases in expression of CYP27A1 mRNA in hGF and hPDLC following incubation with IL-1β or *Pg*-LPS were demonstrated (Fig. 8). By contrast, sodium butyrate did not influence significantly CYP27A1 mRNA expression in hGF and hPDLC (Fig. 8). In addition, no significant differences between hGF and hPDLC were observed in the regulation of CYP27A1.

## Discussion

In the present study, our hypothesis that hGF and hPDLC have 25-hydroxylase activity, and that they can synthesize 25OHD_3_ was verified. Therefore, the origin of high 25OHD_3_ concentrations in gingival crevicular fluid [27], [28] might be hGF and hPDLC. Having demonstrated 1α-hydroxylase activity in hGF and hPDLC [29], we could consider that the conversion of vitamin D_3_ to 1,25OH_2_D_3_ in hGF and hPDLC consisted of two steps: 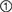 from vitamin D_3_ to 25OHD_3_, under the action of 25-hydroxylase CYP27A1; 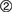 from 25OHD_3_ to 1,25OH_2_D_3_, under the action of 1α-hydroxylase CYP27B1. This two-step conversion is similar to that observed in human keratinocytes [7], [19], [30], [31], [32]. In addition, Slominski et al. reported an alternate pathway of vitamin D_3_ metabolism by cytochrome P450scc (CYP11A1) [33], [34], [35], [36]. P450scc activity in hGF and hPDLC is worth further investigation in our future study.

We can then calculate and compare the amount of 1,25OH_2_D_3_ synthesized from 1000 nM vitamin D_3_ and from 1000 nM 25OHD_3_. According to the present study, the amount of 1,25OH_2_D_3_ generated would be: 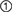 In hGF exposed to 1000 nM vitamin D_3_ for 48 h, 9 fmol/10000 cells in supernatants +14 fmol/10000 cells in cell lysates = 23 fmol/10000 cells (Fig. 4). 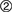 In hPDLC exposed to 1000 nM vitamin D_3_ for 48 h, 13 fmol/10000 cells in supernatants +16 fmol/10000 cells in cell lysates = 29 fmol/10000 cells (Fig. 4). According to our previous study [29], the amount of 1,25OH_2_D_3_ generated would be the following: 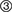 In hGF exposed to 1000 nM 25OHD_3_ for 48 h, 5 fmol/10000 cells in supernatants +13 fmol/10000 cells in cell lysates = 18 fmol/10000 cells. 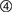 In hPDLC exposed to 1000 nM 25OHD_3_ for 48 h, 13 fmol/10000 cells in supernatants +14 fmol/10000 cells in cell lysates = 27 fmol/10000 cells. It is interesting that 1000 nM vitamin D_3_ could induce hGF and hPDLC to generate even more 1,25OH_2_D_3_ than 1000 nM 25OHD_3_. Particular attention should be paid to the observation that after 1000 nM vitamin D_3_ treatment for 48 h, the 25OHD_3_ concentration in the cell supernatants of hGF and hPDLC were only about 45 nM–64 nM and 30 nM–50 nM respectively, much lower than the added 1000 nM vitamin D_3_. So, why was less 25OHD_3_ converted to more 1,25OH_2_D_3_? One reason might be that after vitamin D_3_ treatment, 25OHD_3_ is found not only in the supernatant, but also in the cell lysates, allowing intracellular 25OHD_3_ to act directly as substrate of 1α-hydroxylase. On the other hand, exogenous 25OHD_3_ should enter the cells before eliciting a response. Thus, the direct availability at the site of action might be of great importance.

After comprehensive verification of 25-hydroxylase activity and the demonstration of CYP27A1 as the key 25-hydroxylase in hGF and hPDLC, the regulation of CYP27A1 in these cells was preliminarily investigated. IL-1β in gingival crevicular fluids of patients with periodontitis decreases significantly after initial periodontal therapy, indicating that IL-1β is associated with periodontitis [28]. *Porphyromonas gingivalis* is an important pathogen of periodontitis and butyrate is one of its metabolites [37]. It was demonstrated that the butyrate concentrations in gingival crevicular fluids of patients with periodontitis are significantly higher than those of healthy controls, and that butyrate concentrations in gingival crevicular fluids are significantly correlated with periodontal inflammation [38], [39]. To investigate the regulation of CYP27A1 in hGF and hPDLC, IL-1β, *Pg*-LPS and sodium butyrate were chosen for the present study. It should be considered, however, that although stimuli with periodontal characteristics were used to simulate a periodontitis-like condition, this does not properly model the chronic disease situation *in vivo*, and can only help to investigate the regulation of CYP27A1 in hGF and hPDLC. The NF-κB activator, IL-1β, was demonstrated to be a potent up-regulator of CYP27A1 mRNA in hGF and hPDLC (Fig. 8). *Pg*-LPS could also up-regulate significantly the expression of CYP27A1 mRNA, whereas sodium butyrate could not. It was reported that *Pg*-LPS is the ligand of Toll-like receptor 2 (TLR2) and TLR4 [40], [41] and that both hGF and hPDLC expressed TLR2 and TLR4 [42]. Upon ligand binding, TLR2 or TLR4-mediated signaling could activate signal transduction, leading to NF-κB activation [43], [44]. Thus, NF-κB might be involved in the regulation of CYP27A1 expression, an observation that warrants further investigation.

Each donor supplied both hGF and hPDLC in the present study. Although hGF and hPDLC are two different kinds of cells, they shared many features in 25-hydroxylase expression, activity and regulation, and only subtle differences were detected. As shown in Fig. 6, when CYP2R1 was knocked down, 25OHD_3_ generation by hGF was not changed significantly, whereas 25OHD_3_ generation by hPDLC was affected slightly. However, the difference did not affect our conclusion that CYP27A1 might be the key 25-hydroxylase in hGF and hPDLC.

Since 1,25OH_2_D_3_ may enhance the antibacterial defense of human gingival epithelial cells [45] and hGF and hPDLC could synthesize 1,25OH_2_D_3_ with 25OHD_3_ [29], the confirmation of 25-hydroxylase activity in hGF and hPDLC implies that these cells could generate 25OHD_3_ as a substrate for 1,25OH_2_D_3_. From this perspective, 25-hydroxylase activity in hGF and hPDLC may be involved in the innate immune defense of the oral cavity. Recently, it was reported that oral calcium and vitamin D supplementation have a positive effect on periodontal health [46], [47]. However, topical application of vitamin D has not been reported. Since hGF and hPDLC have the ability to synthesize 25OHD_3_ and then to synthesize 1,25OH_2_D_3_, the topical application of vitamin D_3_ might fulfill the function of 1,25OH_2_D_3_. Thus, our data suggest a potential benefit of topical application of vitamin D_3_ in periodontal therapy.

In conclusion, hGF and hPDLC were identified as new extra-hepatic sites of 25OHD_3_ synthesis for the first time, and CYP27A1 might be the key 25-hydroxylase in these cells.

## Materials and Methods

### Ethics Statement

The study protocol was approved by the institutional review board of Peking University School and Hospital of Stomatology (PKUSSIRB-2011007) and written informed consent was obtained from each participant in accordance with the Declaration of Helsinki.

### Cell Culture

Primary culture of hGF and hPDLC was carried out according to our previous methods [29]. In brief, hPDLC were obtained from extracted third molars of 5 young healthy volunteers, and hGF was isolated from the gingiva of the same 5 donors. The periodontal ligament tissues attached to the middle third of the roots were curetted gently by a surgical scalpel, minced and placed in 24-well plates. Gingivae were also minced and transferred into 24-well plates. Tissue explants were cultured in Dulbecco’s Modified Eagle’s Medium (DMEM; Gibco, Grand Island, NY, USA) supplemented with 10% (v/v) fetal bovine serum (FBS; PAA, Coelbe, Germany), 100 U/mL penicillin G and 100 µg/mL streptomycin. Cultures were maintained in a humidified atmosphere of 5% (v/v) CO_2_ at 37°C. After reaching 80% confluence, hGF and hPDLC were digested with a mixture of 0.25% (w/v) trypsin and 0.02% (w/v) EDTA, and subcultured at a 1∶3 ratio. DMEM without phenol red (Sigma, St. Louis, MO, USA), 10% (v/v) dextran-coated, charcoal-stripped FBS (DCC-FBS; TBD, Tianjin, China) and hGF and hPDLC of passage 4 were used in all the following experiments. All experiments were conducted in triplicate.

The prostate cancer cell line, PC-3 (American Type Culture Collection, Rockville, MD, USA), was cultured in RPMI 1640 (Gibco, Gaithersburg, MD, USA) supplemented with 10% (v/v) FBS (FBS; PAA, Coelbe, Germany) in a humidified atmosphere of 5% CO_2_ at 37°C and was used when the cells were in the logarithmic phase and reached 80% confluence.

### Cytotoxicity Test of Vitamin D_3_

hGF and hPDLC of three donors were used in the cytotoxicity test. hGF and hPDLC in their logarithmic growth phase were plated into 96-well plates at a density of 3000 cells/well in DMEM with 10% DCC-FBS, and the medium was replaced by DMEM without DCC-FBS after 24 h. After another 24 h, the medium was changed to DMEM with 10% DCC-FBS, and supplemented with 1000 nM vitamin D_3_ or vehicle, respectively. The cytotoxicity test was carried out according to the Cell Counting Kit-8 protocol (CCK-8; Dojindo, Kumamoto, Japan). At hours 0, 24 and 48, cells were incubated with CCK-8 for the last 3 h of the culture period, after which the optical density values (OD values) were detected at 490 nm with a microplate reader (Bio-Rad Model 550, Hercules, CA, USA).

### Detection of 25-hydroxylase Expression

hGF and hPDLC from all five donors were seeded into six-well plates at a density of 5000 cm^−2^ in DMEM supplemented with 10% DCC-FBS. Four days later, a portion of the cells were harvested using Trizol agent (Dongsheng Biotech, Guangzhou, China). RNA was extracted using Trizol according to the manufacturer’s instructions, and was reverse transcribed to cDNA using a reverse transcription kit (Bio-Rad, Hercules, CA, USA). Real-time PCR reactions were accomplished using SYBR® Premix Ex Taq™ II (TaKaRa Biotechnology, Dalian, China) in an ABI 7500 real-time Thermocycler (Applied Biosystems, Foster City, CA, USA). The data were analyzed using the SDS software, according to the manufacturer’s instructions.

Glyceraldehyde-3-phosphate-dehydrogenase (GAPDH) was used as an internal control. Data were presented as relative mRNA levels calculated by the equation 2^−ΔCt^ (ΔCt = Ct of target gene minus Ct of GAPDH) [48]. The primers used are listed in Table 1.

**Table 1:** Primer sequences used for PCR or real-time PCR.

Target genes	Forward primer (5′ →3′)	Reverse primer (5′ →3′)	Products (bp)
CYP27A1	GCTCTTGGAGCAAGTGATG	AGCATCCGTATAGAGCGC	196
CYP2R1	TTGGAGGCATATCAACTGTGGT	CTCGGCCATATCTGGAATTGAG	153
GAPDH	GAAGGTGAAGGTCGGAGTC	GAAGATGGTGATGGGATTTC	226

PC-3 cells and the remaining hGF and hPDLC were harvested using lysis buffer [20 mM Tris (pH 7.4), 150 mM NaCl, 1 mM EDTA, 1 mM EGTA, 1% (v/v) Triton X-100, 2.5 mM sodium pyrophosphate, 1 mM β-glycerol phosphate and 2 mM Na_3_VO_4_ supplemented with a protease inhibitor cocktail (Roche, Mannheim, Germany)] [29] for Western blotting. The protein concentration was determined using the Bicinchoninic Acid Protein Assay Kit (Applygen, Beijing, China). Twenty micrograms of total protein from each sample were loaded onto a gel comprising a 5% (w/v) stacking gel and a 10% (w/v) running gel. At the end of the electrophoresis, samples were transferred onto nitrocellulose blotting membranes (Hybond™; Amersham Pharmacia, Little Chalfont, UK). Blots were probed with a goat polyclonal antibody to CYP27A1 (diluted 1∶200; Santa Cruz Biotechnology, Santa Cruz, CA, USA), a mouse polyclonal antibody to CYP2R1 (diluted 1∶500; ABCAM, Cambridge, UK) or a mouse monoclonal antibody to β-actin (diluted 1∶1000; Santa Cruz Biotechnology, Santa Cruz, CA, USA). After washing, blots were incubated with horseradish peroxidase-linked secondary antibody. The secondary antibodies against sheep (Kirkegaard & Perry Laboratories, Inc., Maryland, USA) and mouse (Beijing Zhongshan Golden Bridge Biotechnology, Beijing, China) IgG were both diluted 1∶2500. Antigen-antibody complexes were detected using the Enhanced Chemiluminescence reagent (Applygen, Beijing, China).

### Detection of 25OHD_3_ Production

Cells from 3 donors were treated with 1000 nM vitamin D_3_ (Sigma, St. Louis, MO, USA) for 1, 4, 12, 24 or 48 h, after which supernatants were collected, and the cells were scraped in PBS containing 0.2% Triton X-100 and stored at −80°C. Prior to use, cell lysates were sonicated on ice in a sonifier cell disrupter for 2×15 s. The levels of 25OHD_3_ in cell supernatants and cell lysates were detected using a 25OHD_3_ radioimmunoassay kit (DiaSorin, Stillwater, MN, USA) with a sensitivity of 1.5 ng/mL.

### Detection of 1,25OH_2_D_3_ Production

Cells from 3 donors were treated with 1000 nM vitamin D_3_ (Sigma, St. Louis, MO, USA) for 48 h and then supernatants were collected and cells were scraped in PBS containing 0.2% Triton X-100 and stored at −80°C. Prior to use, cell lysates were sonicated on ice in a sonifier cell disrupter for 2×15 s. The levels of 1,25OH_2_D_3_ in cell supernatants and cell lysates were determined using a 1,25OH_2_D_3_ radioimmunoassay kit (DiaSorin, Stillwater, MN, USA). The sensitivity of the assay was 2.0 pg/mL.

### RNA Interference of 25-hydroxylase

To confirm the dependence of vitamin D_3_ conversion to 25OHD_3_ on 25-hydroxylase, the highly specific technique of RNA interference was utilized. Cells were seeded at a density of 15000 cm^−2^ in six-well plates. Eight hours later, the cells were transfected with either CYP27A1 siRNA (10 nM) or CYP2R1 siRNA (10 nM), or a non-silencing control siRNA using Hiperfect™ transfection reagent (Qiagen, Duesseldorf, Germany), according to the manufacturer's instructions. The target sequence of CYP27A1 siRNA was 5′- CACGCTGACATGGGCCCTGTA -3′, the target sequence of CYP2R1 siRNA was 5′- TGGGTTGATCACAGACGATTA -3′, and the non-silencing control was a non-homologous, scrambled sequence equivalent.

Sixty hours after transfection, cells were harvested, RNA and cDNA were obtained, and real-time PCR was performed as described earlier to test the effect of RNAi.

After confirming the effect of RNAi, 25OHD_3_ production after RNAi was determined. Cells were first transfected with CYP27A1 siRNA (10 nM) or CYP2R1 siRNA (10 nM), or non-silencing control siRNA. Twelve hours after transfection, these cells were treated with 100 nM, 200 nM, 400 nM, 600 nM or 1000 nM vitamin D_3_ (Sigma, St. Louis, MO, USA) for another 12 h. Then, the 25OHD_3_ concentrations in the cell supernatants and cell lysates were determined as described earlier.

Some other cells were first transfected with CYP27A1 siRNA (10 nM), or non-silencing control siRNA, and 12 h after transfection, these cells were treated with 1000 nM vitamin D_3_ (Sigma, St. Louis, MO, USA) for another 48 h. Then, the 1,25OH_2_D_3_ concentrations in the cell supernatants and cell lysates were detected as described earlier.

### Regulation of CYP27A1 in hGF and hPDLC

Cells from four donors were seeded into six-well plates at a density of 5000 cm^−2^ in DMEM supplemented with 10% DCC-FBS. Four days later, cells were incubated with IL-1β (PeproTech, London, UK; 1 ng/mL and 10 ng/mL), *Pg*-LPS (Invivogen, San Diego, CA, USA; 1 µg/mL and 10 µg/mL) or sodium butyrate (SCRC, Shanghai, China; 4 mM) for 24 h. Then mRNA expression was detected by real-time PCR as described previously.

### Statistical Methods

The Shapiro-Wilk test was used to determinate the distribution of the variants. The paired samples t-test was used to compare differences of the mRNA expression levels of CYP27A1 and CYP2R1 between hGF and hPDLC, differences of 25OHD_3_ generation by hGF and hPDLC, and the effect of RNA interference. Comparison of 25OHD_3_ generation with and without knockdown of 25-hydroxylase, and 1,25OH_2_D_3_ generation with and without knockdown of CYP27A1 were also performed using a paired samples t-test. The impact of stimulation on CYP27A1 mRNA expression was analyzed using a paired-samples t-test, and the difference between CYP27A1 regulation in hGF and hPDLC was analyzed using a Wilcoxon test.

Statistical analyses were accomplished using the SPSS 11.5 software package (SPSS Inc., Chicago, IL, USA). A *p* value <0.05 was considered statistically significant.
